# The Analysis of Sialylation, *N*-Glycan Branching, and Expression of *O*-Glycans in Seminal Plasma of Infertile Men

**DOI:** 10.1155/2015/941871

**Published:** 2015-03-29

**Authors:** Ewa M. Kratz, Anna Kałuża, Mariusz Zimmer, Mirosława Ferens-Sieczkowska

**Affiliations:** ^1^Department of Chemistry and Immunochemistry, Wrocław Medical University, Bujwida 44a, 50-345 Wrocław, Poland; ^2^2nd Department and Clinic of Gynaecology, Obstetrics and Neonatology, Wrocław Medical University, Academic Hospital, Borowska 213, 50-556 Wrocław, Poland

## Abstract

Carbohydrates are known to mediate some events involved in successful fertilization. Although some studies on the glycosylation of seminal plasma proteins are available, the total glycan profile was rarely analyzed as a feature influencing fertilization potential. In this work we aimed to compare some glycosylation traits in seminal plasma glycoproteins of fertile and infertile men. The following findings emerge from our studies: (1) in human seminal plasma the presence and alterations of *O*-linked glycans were observed; (2) the expression of SNA-reactive sialic acid significantly differs between asthenozoospermia and both normozoospermic (fertile and infertile) groups; (3) the expression of PHA-L-reactive highly branched *N*-glycans was significantly lower in oligozoospermic patients than in both normozoospermic groups. Indication of the appropriate lectins that would enable the possibly precise determination of the glycan profile seems to be a good supplement to mass spectrum analysis. Extension of the lectin panel is useful for the further research.

## 1. Introduction

Although many molecular events preceding direct fusion of gametes have been well described, andrologists still wonder what the impact of protein composition and posttranslational modifications in the seminal plasma on the success or failure of fertilization is. Male gametes remain suspended in seminal plasma for a relatively short time, as most of its components are unlikely to pass through the cervix [1, 2]. The main role of seminal plasma proteins is thought to maintain sperm in a decapacitated state, hold in cervical os, and protect from harsh environment of vagina [1, 2]. Nevertheless, some researchers claim that components of seminal plasma may assist sperm in penetrating cervical mucus border, as it was shown in some animal species [1, 3–5]. So far, information on the molecular nature of such possible interaction is not available.

In the recent years increasing number of couples facing conceiving problems stimulate intensive search for the reasons of decreased male fertility. Standard semen analysis, comprising sperm count, motility, morphology, and vitality, seems to be insufficient as a predictive factor, and thus the need to establish new biomarkers, helpful in estimation of male fertility, becomes seriously challenging [6–8].

It has been shown that the sugar moieties of the seminal plasma glycoproteins contain some peculiar glycans, which are rarely present under normal, physiological conditions, in the tissues and other body fluids of healthy people. The obligatory presence of high-mannose type oligosaccharides was proven for seminal glycodelin [9] and was suggested also for PSA (prostate specific antigen) and PAP (prostate alkaline phosphatase) [10, 11]. Apart from the expression of terminal mannose, additional lactosamine units and GalNAc presence in the* N*-glycans, lack of sialic acid in a significant number of glycans, extremely high fucose content, and substantial branching of some* N*-glycans have been reported (reviewed in [12–14]). These structural features are mentioned not only in the work of Pang et al. [15], the only authors so far who presented detailed composition of human seminal plasma glycome, but also in the reports concerning glycosylation of particular glycoproteins, like PSA or PAP, and associated with the diseases of male reproductive tract [11, 16–18]. On the other hand, also in our earlier studies, some alterations of the glycosylation profile were found to be associated with fertility status. Lower degree of seminal fibronectin sialylation was associated with abnormal semen parameters of infertile men [19, 20]. Increased fucosylation was found in a number of glycoproteins [21], while differences in antennary fucose expression were detected in IgG and secretory component of IgA [22, 23]. In the mentioned above study of Pang et al. [15] the seminal plasma glycome was studied in only 4 samples of the fertile men. The intersubject variability of oligosaccharides was substantial; thus, the comparison of healthy and diseased samples must enroll much larger groups of samples. This, however, seems to be difficult by means of mass spectrometry.

Our aim was thus to learn if some so far described features of glycan structure may be altered in seminal plasma glycome of infertile men and, also, if such potential alterations may be detected and measured by means of their lectin reactivity. Apart from the lectins commonly used for such purposes, dedicated for sialylation analysis, we also attempted studies of glycan branching with PHA-L (*Phaseolus vulgaris* leucoagglutinin) and expression of complete and truncated* O*-glycans with MPL (*Maclura pomifera* lectin) and VVL (*Vicia villosa* lectin), respectively.

## 2. Materials and Methods

### 2.1. Clinical Samples

The seminal plasma samples were derived from men living in childless couples (at least one year of unprotected intercourse without achieving pregnancy), visiting the Department of Gynaecology, Obstetrics and Neonatology of Wrocław Medical University to perform intrauterine insemination. For the preparation of the ejaculate for insemination, to each sample the equal volume of buffered saline Earle's solution was added to improve sperm motility. To increase sperm concentration, before the introduction of semen to the female reproductive tract, spermatozoa were gently centrifuged (400 ×g, 10 min at room temperature), and to the pellet 1 mL of Earle's solution was added. The supernatant (seminal plasma diluted 1 : 1 with Earle's solution) was collected by decantation. This fraction (volume depending on the sample) constituted the material for our investigations.

The samples were obtained from 131 childless men 25–46 years old and based on the results of standard semen analysis (volume, pH, morphology, sperm concentration, motility, and viability) according to WHO criteria [24], divided into the following groups: normozoospermic infertile (normal semen parameters, 67 patients), asthenozoospermic (with a total motility of <40% or progressive motility <32% at 1 h after ejaculation; 27 patients), oligozoospermic (sperm number <15 × 10^6^/mL of the ejaculate, 15 patients), and oligoasthenozoospermic (reduced number and abnormal sperm motility, 22 patients). The control group enrolled fertile normozoospermic volunteers with proven fertility (they have at least one child; 25–46 years old; 38 subjects). In the normozoospermic fertile and infertile samples the number of spermatozoa was higher than 15 × 10^6^/mL of the ejaculate and more than 4% expressed the normal sperm morphology with a total motility of ≥40% or progressive motility ≥32% at 1 h after ejaculation. Standard semen analysis was performed at the Department of Laboratory Diagnostics, Academic Hospital, Wrocław Medical University. All samples were collected after obtaining patients' informed consent, and the study obtained Wrocław Medical University Bioethics Committee approval (KB-631/2012).

### 2.2. Determination of Seminal Plasma Glycans by Lectin-ELISA

Total expression of carbohydrate epitopes was measured with the direct lectin-ELISA method as described by us previously for fucose [25, 26]. The glycoproteins sialylation was determined using two biotinylated lectins:* Maackia amurensis* agglutinin, MAA and* Sambucus nigra* agglutinin, SNA, recognizing sialic acid *α*2,3- and *α*2,6-linked, respectively (Vector Laboratories Inc., Burlingame, CA, USA). Expression of* O*-glycans was studied with biotinylated lectins:* Maclura pomifera* lectin, MPL and* Vicia villosa* lectin, VVL (Vector Laboratories Inc., Burlingame, CA, USA), detecting complete* O*-glycans and their forms truncated to single GalNAc (Tn antigen), respectively. Expression of highly branched (tri- and tetra-antennary)* N*-glycans was measured with* Phaseolus vulgaris* leucoagglutinin, PHA-L (Vector Laboratories Inc., Burlingame, CA, USA), specific for *β*1,6-linked GlcNAc that occurs in glycans at the third antennae [27, 28].

The wells of the microtiter plates were coated with seminal plasma samples containing 800 ng of protein in 10 mM TBS, pH 7.5, and incubated overnight at room temperature. After washing with TBS, the free binding sites were blocked with 1% BSA solution in TBS (2 h at RT). Then the plates were incubated with biotinylated lectins (1 h, 37°C) diluted with 10 mM TBS-T as follows: MAA 1 : 500, SNA 1 : 1000, MPL 1 : 1000, VVL 1 : 1000, and PHA-L 1 : 500. Next, the plates were incubated with phosphatase-labelled ExtrAvidin (Sigma Chemical Co., St. Louis, MO, USA) for 30 min at 37°C and diluted with TBS-T, 1 mM CaCl_2_, 1 mM MgCl_2_, 1 : 20 000 for MAA, SNA, MPL, and VVL or 1 : 10 000 for PHA-L. After the incubation the phosphatase reaction was developed with p-nitrophenyl phosphate as a substrate. The reaction was stopped with 100 *μ*L of 1 M NaOH per well and the absorbance was read at 405 nm, reference filter 630 nm with a StatFax 200 microplate reader (Awareness Technology Inc., Palm City, FL, USA). After each incubation step the plates were extensively washed with TBS-T (TBS containing 0.05% Tween-20, pH 7.5). All samples were analysed in duplicate. Background absorbance was measured for samples in which all reagents were present, but seminal plasma was replaced with TBS-T. Lectins relative reactivity was expressed in absorbance units (AU). The background absorbance values were not higher than 0.2 AU. The samples were blinded at the moment of assay, because they were coded consecutively at the moment of enrolment, without any respect to the diagnostic group, and they were run in a random order with respect to the groups.

### 2.3. Statistical Analysis

Statistical analysis was performed using the statistical software STATISTICA 10.0 (StatSoft Inc., Tulsa, OK, USA). Experimental data were presented as means and standard deviations (SD), and distribution of the values within analysed groups was presented as box-whisker plots with median and interquartile (25th–75th percentile) range. According to a Shapiro-Wilk *W* test, the values did not fit normal distribution; thus the nonparametric Kruskall-Wallis ANOVA test was used to determine differences among the groups. The correlation with 95% of confidence interval was estimated according to the Spearman test. Statistical significance was accepted for a two-tailed *P* value of less than 0.05.

## 3. Results

**Figure 1:**
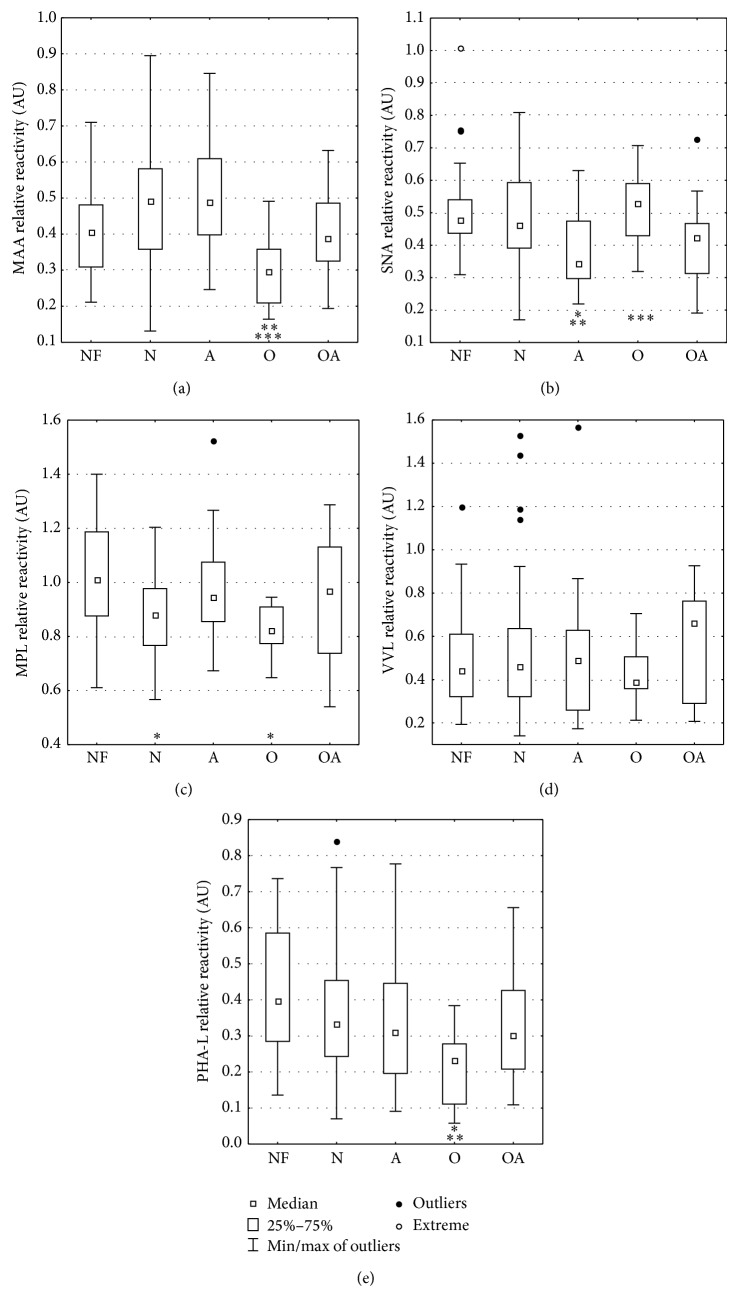
The relative reactivity of seminal glycoproteins with lectins. The results are expressed in absorbance units (AU) as median values of relative reactivities with lectins. Seminal glycoproteins' lectin reactivities were estimated by direct lectin-ELISA and expressed in absorbance units (AU). Seminal groups: NF: normozoospermic fertile, N: normozoospermic infertile, A: asthenozoospermic, O: oligozoospermic, OA: oligoasthenozoospermic. MAA:* Maackia amurensis* agglutinin, SNA:* Sambucus nigra* agglutinin, MPL:* Maclura pomifera* lectin, VVL:* Vicia villosa* lectin, and PHA-L:* Phaseolus vulgaris* leucoagglutinin. For lectin specificity see Section 2. Significant differences versus ^*^normozoospermic fertile, ^**^normozoospermic infertile, and ^***^asthenozoospermic groups. Statistical significance of the differences was accepted for a *P* value of less than 0.05.

Reactivity of seminal plasma glycoproteins with a selected panel of lectins is presented as a mean absorbance and standard deviation in Table 1 and the distribution of values is presented in Figure 1. The only one out of five studied lectins that expressed no statistically significant differences among the patients' groups was VVL, specific for truncated* O*-glycans limited to the single GalNAc (Tn antigen). Total expression of* O*-glycans occurred diminished in two groups: normozoospermic infertile and oligozoospermic subjects. Oligozoospermic seminal plasma samples were also affected by altered expression of sialic acid. Decrease of MAA-reactive, thus *α*2,3-linked sialic acid (SA), was significant when compared to normozoospermic infertile and asthenozoospermic groups, while comparison of *α*2,6-linked SA of oligozoospermic and asthenozoospermic groups showed an increase of the lectin reactivity in the former one. In asthenozoospermic seminal plasma decrease of SNA-reactive SA was observed in comparison with both, fertile and infertile, normozoospermic groups.

**Figure 2:**
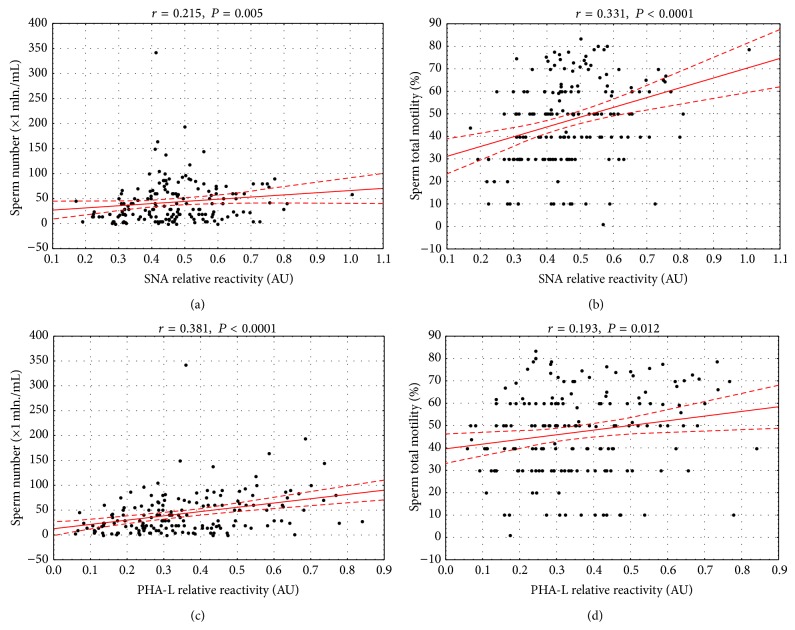
The association between seminal glycoproteins' relative reactivity with SNA and PHA-L and sperm number and their total motility. The figures show the association between glycoproteins' lectin reactivities and sperm number (×1 mln./mL) and their total motility (%): (a) and (b) for SNA, respectively, and (c) and (d) for PHA-L, respectively. Glycoproteins' lectin reactivities were estimated in direct lectin-ELISA and expressed in absorbance units (AU). The correlations were estimated according to the Spearman test and a two-tailed *P* value of less than 0.05 was considered statistically significant. The dashed line shows 95% of confidence interval. SNA:* Sambucus nigra* agglutinin, PHA-L:* Phaseolus vulgaris* leucoagglutinin. For lectin specificity see Section 2.

Spearman correlation factors were close to 0.2, thus suggesting possible weak correlation between MPL and MAA (*r* = 0.206, *P* = 0.0072) and SNA and PHA-L reactivity (*r* = 0.193, *P* = 0.012). Weak but pronounced correlation was found between both* O*-glycan specific lectins (MPL and VVL, *r* = 0.316, *P* < 0.0001). When both sialic acid specific lectins were analyzed, the values were calculated as *r* = −0.152 and *P* = 0.048. The Spearman rank test was also used to estimate association of lectin reactivity of seminal plasma glycoproteins with semen parameters obtained in the standard examination. Among the lectins tested only SNA and PHA-L were found to be related to sperm count and motility (Figure 2). Profound glycan branching and *α*2,6 SA expression positively correlated with the sperm count and motility. Although the correlations should be regarded as weak, the *P* values determined in Spearman rank test, much lower than 0.05, make them reliable.

## 4. Discussion

The role of seminal plasma proteins in successful fertilization still remains in question in humans. Researchers lack experimental data that could shed some light on this problem; however, it is interesting to pay some attention to the conclusions coming from animal studies. Studying ram spermatozoa, Rickard et al. [5] concluded that exposure of epididymal spermatozoa to seminal plasma had a beneficial effect on their ability to traverse the cervix. Interestingly, although there was no direct impact on sperm motility, the migration through the cervical mucus was significantly improved. Suarez and Pacey [1] suggested that one of the roles of seminal plasma could be assisting cervical migration of spermatozoa. Opposite to the mentioned data, detrimental effect of the exposure of spermatozoa to seminal plasma proteins on the final effect of fertilization was also reported [4, 29, 30]. So far the molecular nature of this support has not been elucidated. As protein-carbohydrate crosstalk is a common regulatory mechanism, the question arises if possible alterations in the glycosylation profile may influence the interactions aimed at maintaining sperm vitality and assistance of cervix entrance. The unique features of seminal plasma glycosylation make this approach promising.

In our earlier studies we have found some alterations of sialylation and fucosylation of several seminal plasma glycoproteins that can be associated with the fertility status of men [19–23, 31]. In a more general approach we have also reported that substantially increased fucosylation may be a common feature of many seminal plasma glycoproteins of infertile men, irrespective of their semen parameters [21]. In this study we aimed to obtain a general look at a total expression of selected glycosylation features, to learn if the general pattern of oligosaccharides decorating seminal glycoproteins may be associated with the male fertility potential. The authors who studied glycosylation of seminal plasma glycoproteins paid attention to the substantial expression of highly branched tri- and tetra-antennary oligosaccharides, especially in PSA produced in diseased prostate [16–18, 32]. The presence of such structures was confirmed also in this study by means of PHA-L binding. The lectin reactivity was significantly decreased in oligozoospermic compared to normozoospermic (both fertile and infertile) subjects. Oligozoospermic group seems to be the most affected one with respect to altered expression of glycans. Except for VVL, the lectin that did not distinguish analyzed patients at all, the reactivity with the remaining four lectins was capable of discriminating oligozoospermic patients from the normozoospermic fertile (MPL and PHA-L), normozoospermic infertile (MAA and PHA-L), or asthenozoospermic subjects (SNA and MAA). The analysis of lectin coreactivity suggested also that seminal* N*-glycans are highly branched and *α*2,6 sialylated (SNA and PHA-L positive correlation), while* O*-glycans are terminated by *α*2,3-linked SA (MPL and MAA positive correlation). Slightly stronger positive correlation of MPL and VVL reactivity may indicate that when* O*-glycosylation pathway is intensified, some of the glycans remain not elongated after GalNAc attachment to Ser/Thr. On the other hand, the negative correlation between SA-specific lectins may suggest some competition of sialyltransferases for SA acceptor sites in the process of glycosylation.

More interesting was the association of glycan profile with the semen parameters. Positive correlation of *r* = 0.381 and low *P* value between PHA-L relative reactivity and sperm number confirmed the former observation of decreased PHA-L reactivity in the oligozoospermic group. SNA reactivity seems to be related to total sperm motility (*r* = 0.331), which was confirmed by relevant difference observed between both normozoospermic groups and asthenozoospermic patients.

In all the glycosylation studies based on the application of lectins, the results did not reflect the accurate structure of the oligosaccharides. Lectins may be able to bind less preferred but abundant enough structures and also may not be able to bind the glycans which are conformationally hidden in the glycoprotein structure. In spite of this drawback, lectin reactivity reflects the real accessibility of the analysed glycoepitopes in the native microenvironment and thus also their potential for interactions* in vivo* [19, 20, 22].

Glycosylation of seminal plasma proteins is worth more detailed studies that could be helpful in the association of the glycosylation profile with male fertility potential and explanation of the role of glycoprotein sugar moiety in the processes that regulate fertilization. It seems clear that oligosaccharide branching,* O*-glycan expression, and the sialylation extent may be considered as important features that influence maintaining sperm in the condition that enables fusion with the oocyte. Glycosylation difference may be crucial even it concerns glycoprotein which play a direct role in fertilization process, like glycodelin S, for example. Such a crucial change may be hard to detect, or detected as relatively small, in the crude material because of the low concentration of the glycoprotein in focus. It is also possible that the pattern of glycosylation is different in the particular glycoproteins and as a result averaged in the whole bulk of glycoproteins. In the coming studies we focus on the indication of model glycoproteins that would reflect the glycosylation pattern in the best way and thus present more significant difference among the seminal samples which at the same time would be relatively easy for glycosylation analysis. Indication of the appropriate lectins that would enable the possibly precise determination of the glycan profile seems to be a good supplement to mass spectrum analysis. Extension of the lectin panel is useful for further research.

## Figures and Tables

**Table 1:** Relative reactivity of human seminal plasma glycoproteins with lectins.

Groups	Relative reactivity with lectins (AU)				
	MAA	SNA	MPL	VVL	PHA-L
Normozoospermic fertile *n* = 38	0.409 ± 0.120	0.505 ± 0.125	1.030 ± 0.193	0.477 ± 0.225	0.424 ± 0.172

Normozoospermic infertile *n* = 67	0.488 ± 0.159	0.485 ± 0.145	0.879 ± 0.150 ^1^ *P* = 0.0013	0.520 ± 0.284	0.363 ± 0.167

Asthenozoospermic *n* = 27	0.501 ± 0.145	0.380 ± 0.127 ^1^ *P* = 0.0025 ^2^ *P* = 0.012	0.968 ± 0.196	0.497 ± 0.292	0.337 ± 0.169

Oligozoospermic *n* = 15	0.297 ± 0.097 ^2^ *P* < 0.0001 ^3^ *P* = 0.00015	0.509 ± 0.107 ^3^ *P* = 0.014	0.822 ± 0.085 ^1^ *P* = 0.0014	0.423 ± 0.130	0.211 ± 0.110 ^1^ *P* = 0.00027 ^2^ *P* = 0.01

Oligoasthenozoospermic *n* = 22	0.409 ± 0.133	0.407 ± 0.117	0.944 ± 0.226	0.557 ± 0.255	0.313 ± 0.136

The results are expressed in absorbance units (AU) as mean values ± SD. Seminal glycoproteins' lectin reactivities were estimated by direct lectin-ELISA and expressed in absorbance units (AU). MAA: *Maackia amurensis* agglutinin, SNA: *Sambucus nigra* agglutinin, MPL: *Maclura pomifera* lectin, VVL: *Vicia villosa* lectin, and PHA-L: *Phaseolus vulgaris* leucoagglutinin. For lectin specificity, see Section 2. Significant differences versus ^1^normozoospermic fertile, ^2^normozoospermic infertile, and ^3^asthenozoospermic groups. Statistical significance of the differences was accepted for a *P* value of less than 0.05.
