# Corrigendum to “The Analysis of Sialylation, *N*-Glycan Branching, and Expression of *O*-Glycans in Seminal Plasma of Infertile Men”

**DOI:** 10.1155/2015/652781

**Published:** 2015-07-29

**Authors:** Ewa M. Kratz, Anna Kałuża, Mariusz Zimmer, Mirosława Ferens-Sieczkowska

**Affiliations:** ^1^Department of Chemistry and Immunochemistry, Wrocław Medical University, Bujwida 44a, 50-345 Wrocław, Poland; ^2^2nd Department and Clinic of Gynaecology, Obstetrics and Neonatology, Wrocław Medical University, Academic Hospital, Borowska 213, 50-556 Wrocław, Poland

In the paper titled “The Analysis of Sialylation,* N*-Glycan Branching, and Expression of* O*-Glycans in Seminal Plasma of Infertile Men” [1], there was an error in the Acknowledgment, which should be corrected as follows:
